# Resonant Raman Scattering in Boron-Implanted GaN

**DOI:** 10.3390/mi13020240

**Published:** 2022-01-31

**Authors:** Yi Peng, Wenwang Wei, Muhammad Farooq Saleem, Kai Xiao, Yanlian Yang, Yufei Yang, Yukun Wang, Wenhong Sun

**Affiliations:** 1 Research Center for Optoelectronic Materials and Devices, School of Physical Science & Technology, Guangxi University, Nanning 530004, China; 1907401031@st.gxu.edu.cn (Y.P.); 1814404039@st.gxu.edu.cn (W.W.); farooq@tju.edu.cn (M.F.S.); 2007401020@st.gxu.edu.cn (K.X.); kindy789456@126.com (Y.Y.); 17729828140@163.com (Y.Y.); 2 Guangxi Key Laboratory for Relativistic Astrophysics, School of Physical Science & Technology, Guangxi University, Nanning 530004, China

**Keywords:** GaN, Raman spectroscopy, photoluminescence, B-implantation

## Abstract

A small Boron ion (B-ion) dose of 5 × 10^14^ cm^−2^ was implanted in a GaN epilayer at an energy of 50 keV, and the sample was subjected to high-temperature rapid thermal annealing (RTA). The resonant Raman spectrum (RRS) showed a strong characteristic of a photoluminescence (PL) emission peak associated with GaN before B-ion implantation and RTA treatment. The PL signal decreased significantly after the B-ion implantation and RTA treatment. The analysis of temperature-dependent Raman spectroscopy data indicated the activation of two transitions in B-ion-implanted GaN in different temperature ranges with activation energies of 66 and 116 meV. The transition energies were estimated in the range of 3.357–3.449 eV through calculations. This paper introduces a calculation method that can be used to calculate the activation and transition energies, and it further highlights the strong influence of B-ion implantation on the luminesce of GaN.

## 1. Introduction

The growing interest in research on GaN is largely due to its great potential for the development of devices in short-wavelength and high-voltage electronics [1,2,3,4,5,6]. The use of the ion-implantation technique, which allows the reliable and controllable introduction of ions to a defined region and is not limited by solid solubility, is a critical requirement for advances in GaN device technology. GaN epilayers have been implanted with many kinds of ions [7,8,9,10,11,12,13,14,15,16,17,18]. The microstructural, electrical, and optical properties of the ion-implanted GaN epilayers are critical to device performance. Isoelectronic doping using congeneric elements has been reported to reduce defect density, improve material quality, and increase the radiative recombination [19,20]. Boron, which belongs to the same group of elements as gallium, has received little attention in the ion implantation of GaN. The applications of boron-doped and -alloyed GaN have been reported by some research groups [21,22], but the material properties of boron-doped GaN have not been explored in detail.

Raman spectroscopy is a non-invasive technique that is widely used to characterize the structural properties of semiconductors [23]. When the energy of the Raman laser is in close resonance with a particular electronic transition in the material, the associated photoluminescence (PL) emission peak can be observed, in addition to the fundamental Raman modes, their replicas, and combination modes. In this paper, we report the resonance Raman spectra and evolution of PL in B-ion-implanted and rapid thermal annealing (RTA)-treated GaN. In this study, we combined Raman spectroscopy data with calculations to estimate the activation energies and identify the position of inherently weak transitions.

## 2. Materials and Methods

GaN epilayers 3 μm thick were grown on (0001) sapphire substrates using low-pressure metalorganic chemical vapor deposition (MOCVD) with trimethylgallium and NH_3_ as sources and hydrogen and nitrogen as carrier gases. The GaN samples were implanted at room temperature with a dose of 5 × 10^14^ cm^−2^ B ions with an energy of 50 keV. After implantation, RTA was performed at 1100 °C in a N_2_ environment for 40 s to activate impurities and repair the ion-implantation-induced damage [24].

The measurements were carried out using a Zolix Finder One spectrometer manufactured by Beijing Zolix Company with a 325 nm line of a 30 mW KIMMON He-Cd laser. The signals were collected with a charge-coupled device (CCD) array. The Raman spectra were recorded with backscattering geometry from the growth surface with unpolarized light propagated parallel to the *c*-axis. The samples were sealed in a Linkam cold/hot stage with continuous liquid nitrogen flow that allowed temperature control in the range between 77 and 330 K. The laser beam had a diameter of about 1 μm. Simulations of ion implantation relied on commonly used software: the Stopping and Range of Ions in Matter (SRIM). The wurtzite GaN density used in the simulation was 6.15 g/cm^3^. The HRXRD measurements were carried out using Panalytical’s X’Pert3 MRD with a Ge (220) four-crystal monochromator and Cu Kα1 = 1.5406 Å radiation.

## 3. Results and Discussion

**Figure 1:**
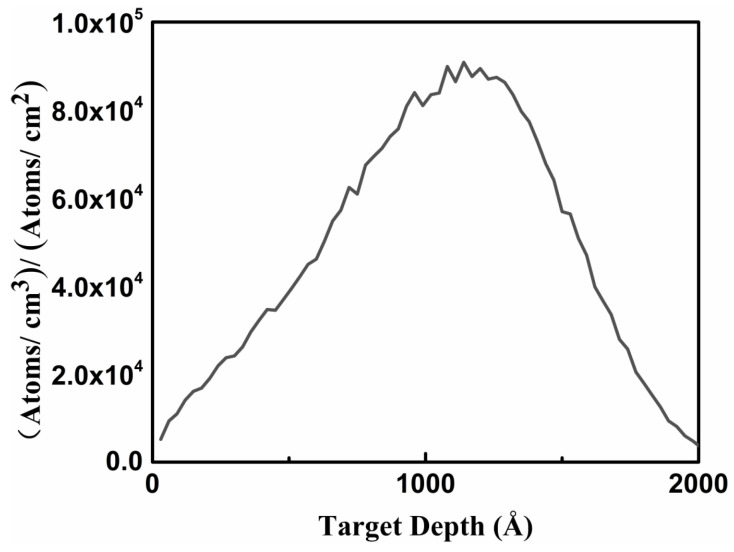
Ion implantation depth of B-implanted GaN simulated by Transport of Ions in Matter.

The result simulated by the Transport of Ions in Matter (TRIM) in the SRIM with an energy of 50 keV at a right angle to the surface is shown in Figure 1, which reveals that the maximum boron-ion-implantation depth in GaN was around 2000 Å, and the implantation depth of maximum ion concentration was 1000 Å with a wurtzite GaN density (6.15 g/cm^3^) of 300 K. According to the ion implantation dose (5 × 10^14^ cm^−2^) and the results of the TRIM, the average concentration of boron was on the level of 10^19^ atoms/cm^3^. Considering the experiment was carried out at a temperature below 300 K, GaN had a smaller absorption coefficient at low temperature [25]. The 325 nm Raman spectrum successfully detected the implantation depth of the maximum ion implantation concentration, or even deeper, based on the research showing that the penetration depth of the 325 nm (3.82 eV) laser light is about 100 nm in GaN [26], meaning more than 50% of the sample can be detected, which had little influence on the Raman results. The boron content was essentially in the same order of magnitude as the whole range, which was at the level of 10^19^ atoms/cm^3^. It was much smaller than the value of gallium at 5 × 10^22^ atoms/cm^3^ in wurtzite GaN, which indicates it is nearly impossible to form an alloy. This was supported by the HRXRD results of the lattice parameter. The Gaussian distribution of single-time ion implantation is well-known, and a large number of studies on Raman spectra in ion implantation have been conducted under the condition of single-time ion implantation [14,15,27], so the issue of inhomogeneity is not discussed in this paper.

**Figure 2:**
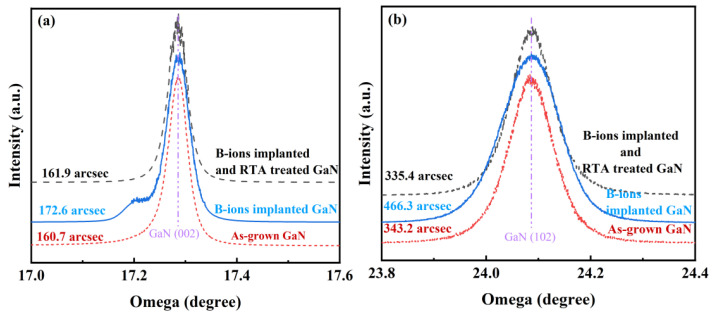
Comparison of (**a**) (002) and (**b**) (102) high-resolution X-ray diffraction rocking curves of the as-grown GaN with B-ion-implanted and rapid thermal annealing treated GaN.

Figure 2 shows the (002) and (102) high-resolution X-ray diffraction (HRXRD) rocking curves of the samples. The full width at half maxima (FWHMs) of the GaN (002) crystal planes in as-grown GaN, as-implanted GaN, and B-implanted and RTA-treated GaN are 160.7, 172.6, and 161.9 arcsec, respectively. The FWHM values of the GaN (102) crystal planes are 343.2, 466.3, and 335.4 arcsec, respectively. This indicates that the post-implantation damage caused by ion implantation was removed from the examined layers and the layer quality was improved after RTA treatment.

**Figure 3:**
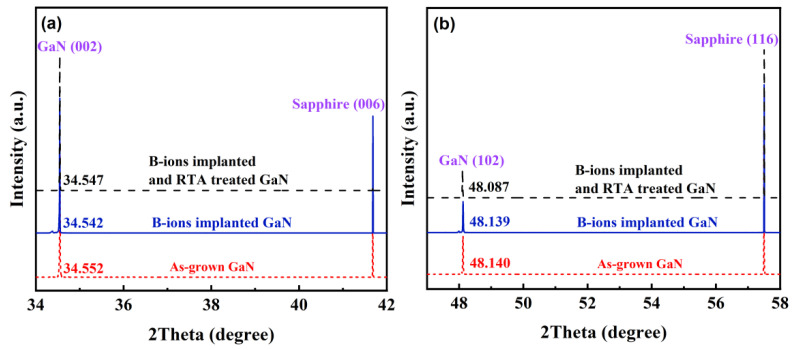
Comparison of (**a**) (002) and (**b**) (102) high-resolution X-ray diffraction 2θ-ω scan of the as-grown GaN with B-ion-implanted and rapid thermal annealing treated GaN.

The lattice constants of the samples were precisely determined by HRXRD symmetric and skew symmetric 2θ-ω scans in Figure 3. Based on the well-known Bragg equation (2dsinθ = nλ), the interplanar distances can be calculated using Equation (1).

(1) $$d_{hkl}=1/\sqrt{\frac{4}{3}\left(\frac{h^{2}+hk+k^{2}}{a^{2}}\right)+\left(\frac{l^{2}}{c^{2}}\right)}\;$$

We calculated the lattice constants of as-grown GaN, as-implanted GaN, and B-implanted and RTA-treated GaN, which are *a_ag_* = 3.182 Å and *c_ag_* = 5.188 Å, *a_ai_* = 3.181 Å and *c_ai_* = 5.189 Å, and *a_RTA_* = 3.188 Å and *c_RTA_* = 5.188 Å, respectively. The horizontal strain *ε_//_* and vertical strain *ε_⊥_* were calculated by the expression as follows: *ε_//_* = (*a* − *a_0_*)/*a_0_*, *ε_⊥_* = (*c* − *c_0_*)/*c_0_*, where *a_0_* and *c_0_* are the lattice constants of bulk wurtzite GaN (*a_0_* = 3.189 Å, *c_0_* = 5.186 Å). The calculated biaxial strain values are *ε_//_* = −0.00227 and *ε_⊥_* = 0.00032, *ε_//_* =−0.00227 and *ε_⊥_* = 0.00060, and *ε_//_* = −0.00227 and *ε_⊥_* = 0.00046, respectively. The results showed that the strain increased after implantation and decreased after RTA treatment, but due to the implantation dose, the strain remained stable in general.

**Figure 4:**
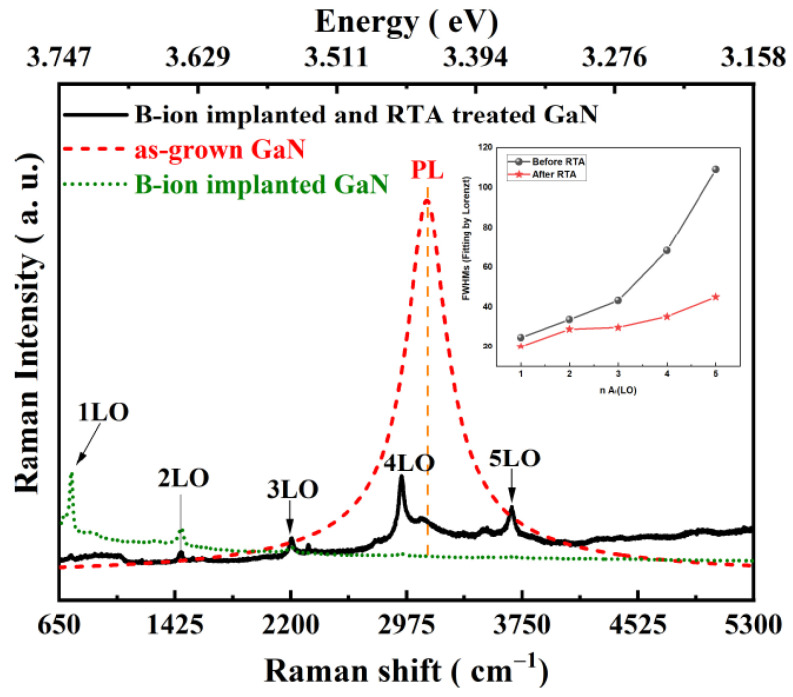
Comparison of the Raman spectra of the as-grown GaN to the B-ion-implanted and rapid thermal annealing treated GaN. The insert is the FWHMs of 1–5 A_1_(LO) Raman peaks before and after the RTA process.

Figure 4 shows a comparison of the room-temperature Raman spectra of the undoped, B-ion implanted, and B-ion-implanted and RTA-treated GaN samples. The GaN shows a strong PL signal of energy 3.429 eV before B-ion implantation. The PL emission decreased after B-ion implantation. The B-ion implantation resulted in the change in electronic transition in GaN. The high PL signal for undoped GaN indicates that the laser was in close resonance to an electronic transition before doping. After RTA treatment, the A_1_(LO) modes were observed up to the fifth order, which were only observed clearly until second order before RTA treatment. Compared to Figure 2, this shows that more distinct resonant Raman peaks were observed after healing post-implantation damage, and their relative intensities changed. This is in agreement with the cascade model postulated by Martin and Varma [14]. The higher-order n-LO lines involve electron transition between the trap levels, which are created by impurities in the forbidden energy gap. The RTA process drives B to migrate to the substitute Ga in GaN, which is why the number of phonon replicas increases and their relative intensities change [28,29]. However, the PL signal was not restored. Therefore, the PL decrease after B-ion implantation indicates that a shift in the associated energy level of transition occurred due to doping. The FWHMs of the Raman peaks before RTA were larger than after RTA, as shown in the insert in Figure 4.

**Figure 5:**
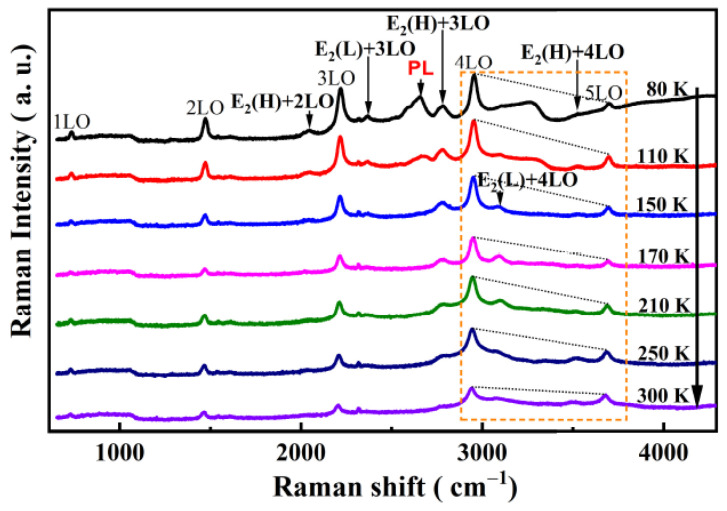
Raman spectra of the rapid thermal annealing treated, B-ion-implanted GaN in the temperature range of 80–300 K. The change in the relative intensity of the 4LO and 5LO phonon modes is discernible in the area in the orange box by observing the change in the slope of the line segment between 4LO and 5LO.

Figure 5 shows the temperature-dependent Raman spectra of the B-implanted and RTA-treated GaN in the temperature range of 80–300 K. A series of peaks appeared that are assigned as A_1_(LO) mode and its multiples. For example, the spectrum acquired at 80 K shows the A_1_(LO) modes from the first to the fifth order at 734, 1473, 2219, 2955, and 3701 cm^−1^, respectively. These results are in agreement with the cascade model [14]. A series of combination modes of nA_1_(LO)+E_2_(high) also appeared at 2047, 2779, and 3526 cm^−1^ for *n* = 1, 2, and 3, respectively. The overtones are two-phonon states that satisfy wave-vector selection rules [30]. The peak at 2319 cm^−1^ is associated with localized impurities [14]. All the peaks fit with the Lorenz shape.

**Figure 6:**
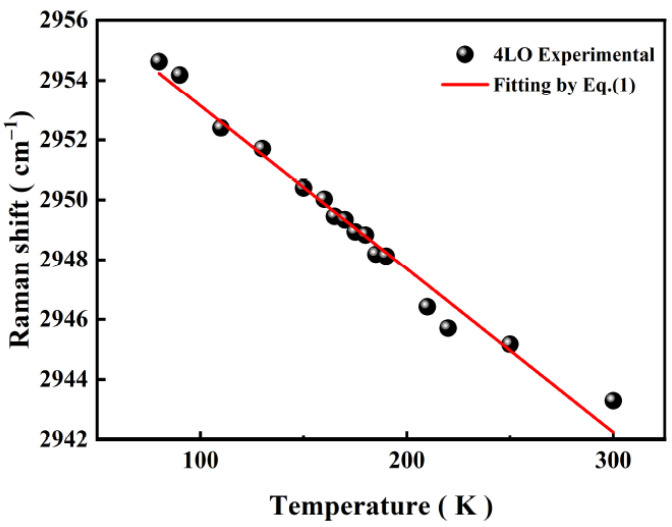
Temperature dependence of the Raman shifts of the 4th-order A_1_(LO) mode in the rapid thermal annealing treated, B-implanted GaN.

The first–fifth-order A_1_(LO) modes were observed at all temperature points in the given range, but the peak positions and relative intensities of each order A_1_(LO) mode changed with temperature. This can be seen in the fourth-order A_1_(LO) mode, which had the strongest signal, and its peak redshifted with temperature, as shown in Figure 6. Two aspects are normally considered responsible for this tendency: lattice thermal expansion and the harmonic effect between phonons [31,32]. A pure empirical equation based on the band gap renormalization by phonon–electron interaction in the Einstein approximation fits this experimental data well [33]:

(2) $$\omega (T)=\omega_{0}-\frac{N}{e^{Mћw_{0}/k_{B}T}-1}\;$$

where *N* and *M* are fitting parameters. Linear fitting was found in our experimental temperature range using Equation (2), and the slope had a value of −0.055 cm^−1^/K for the fourth-order A_1_(LO) mode. These Raman shifts can reflect the change in stress, since the Raman active E_2_ mode comes from atom displacements in the c-plane, and the A_1_ mode relates to the atom’s relative motion along the c-axis. Therefore, the shift in A_1_(LO) suggests the change in stress by comparing it to the stress-free state [33]. The relationship between temperature and stress was established by linear fitting.

**Figure 7:**
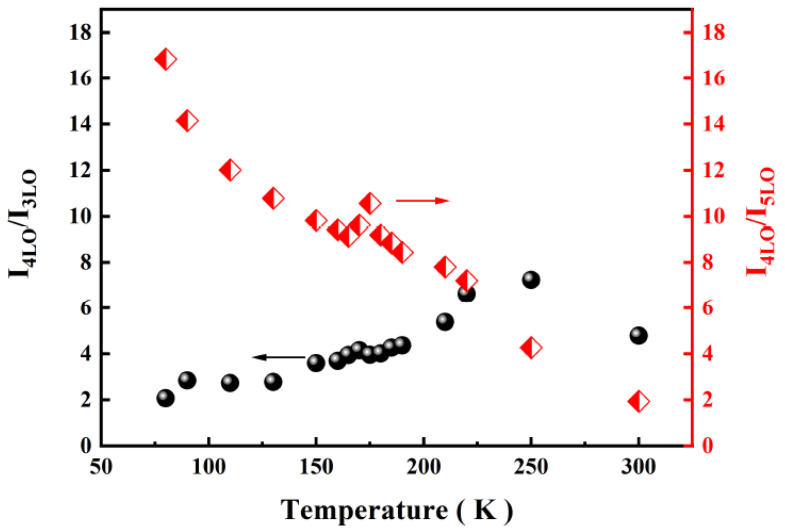
Temperature dependence of integrated intensity ratios of I_4LO_/I_5LO_ and I_4LO_/I_3LO_ for the rapid thermal annealing treated, B-implanted GaN.

The intensity of the fourth-order A_1_(LO) mode was dominant among all A_1_(LO) modes at 80 K and remained almost constant throughout the whole temperature range. However, by observing the change in the slope of the line segment between 4LO and 5LO in the area marked with an orange dotted line in Figure 5, it can be seen that the relative intensity of the fifth-order A_1_(LO) mode increased with the increase in temperature. In Figure 7, the integrated intensity ratios of the fourth- to fifth-order and the third- to fifth-order A_1_(LO) modes are plotted for the B-implanted and RTA-treated sample. The intensity ratios for both the I_4LO_/I_5LO_ and I_4LO_/I_3LO_ were much higher than unity in the whole temperature range, where I_4LO_/I_5LO_ changed more significantly.

It has been reported that the energy of the transition is most enhanced under resonance in the vicinity of the phonon mode [28,34,35,36]. Therefore, the weak signal in the B-ion-implanted sample that appeared between the third-order and the fifth-order A_1_(LO) modes (3.449 ± 0.092 eV, see Figure 5) at 80 K can be assigned as PL. Combining Figure 5 with Figure 7, it is clear that the position of the most enhanced A_1_(LO) mode moved forward in a high wavenumber direction with the temperature increase, i.e., the energy of the transition was in the range of 3.357–3.449 eV.

The bandgap of GaN at different temperatures is obtained using the Varshni empirical equation:

(3) $$E_{0}(T)=E_{0}-\frac{\alpha T^{2}}{\beta +T}$$

where *α* = 2.85 × 10^−4^ eV/K and *β* = 34.01 K, which are fitting parameters [29]. If the value of the low-temperature bandgap of GaN is 3.504 eV [37], the transition energy of the band edge at 80 K is near ~3.488 eV, which is 39–131 meV higher than the energy of the observed PL signal at 3.357–3.449 eV. This suggests that the transition energy of the ion-implanted and RTA-treated sample is less than the energy of the bandgap of GaN.

**Figure 8:**
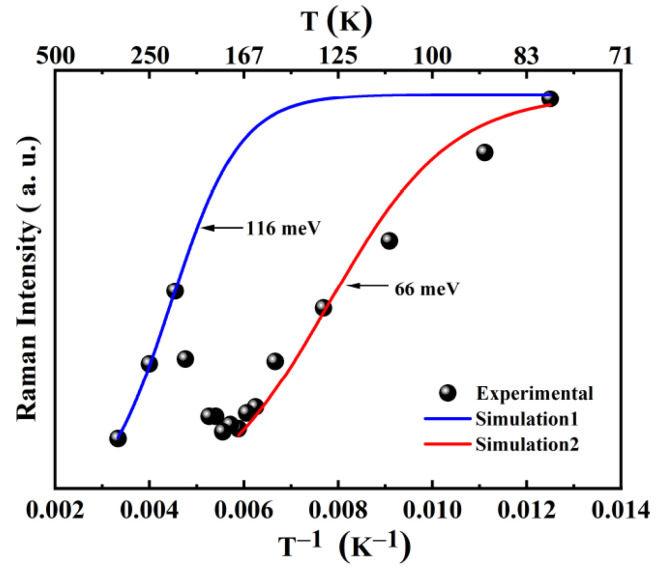
Temperature dependence of the sum of integrated intensities of the first–fifth A_1_(LO) modes for the rapid thermal annealing treated, B-implanted GaN and fitting curve for the estimated activation energies.

Given that all photons measured in the Raman spectra were recorded under the same scattering cross-section, the intensity of the Raman signals should be linearly proportional to the intensity of the PL signal. The sum of the intensities of the first to fifth-order A_1_(LO) modes is plotted against temperature in Figure 8. All the peaks fit the Lorenz shape.

A turning point at 170 K divides the regular plot into two sections. These two different parts indicate that two different transitions predominated in two temperature ranges: 80–170 and 170–300 K. A decreasing trend in intensities was observed below 170 K, and the same trend was observed above 210 K, in agreement with the studies of thermal quenching [38]. However, increased intensity at 170 K points toward the emergence of another recombination channel at this temperature. The two transitions might have originated from different doping positions and charge states of impurity in the lattice.

First-principles studies showed that boron is more likely to substitute Ga upon implantation. In such a case, Boron is mainly in a zero charge state with the presence of a positive charge state over a very small range of Fermi levels near the bottom of the valence band [39]. In order to understand it better, simulation in the two temperature ranges with a single channel dissociation Arrhenius model was carried out [38] using the following equation:

(4) $$I_{T}=\frac{I_{0}}{1+C\exp(-\frac{E_{A}}{kT})}$$

where *I_T_* and *I_0_* are the intensities of the integrated area are obtained by summing up the intensities of the first to fifth A_1_(LO) modes at the temperatures of *T* and 0 K, respectively; *k* is the Boltzmann constant. Fitting the data to Equation (4), the activation energy *E_A_* of the two transitions were obtained as 66 and 116 meV, respectively. The fitting values of *I_0_* and *C* that were obtained by the calculations were 1.7 × 10^6^ and 471, respectively.

**Figure 9:**
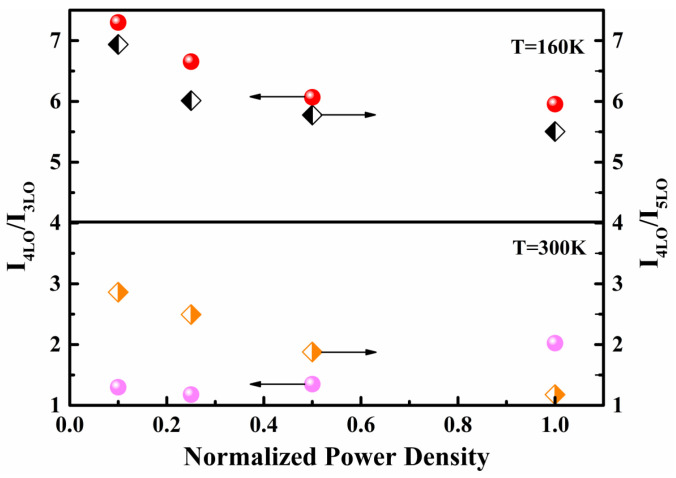
Dependence of ratios between integrated intensities I_4LO_/I_5LO_ and I_4LO_/I_3LO_ on excitation power density at 160 K and 300 K for the rapid thermal annealing treated, B-ion-implanted GaN.

The excitation-power-dependent Raman spectra can also be used to study the type of transition [40]. It was necessary to choose two temperature points below and above 170 K for investigation due to the existence of two transitions. Figure 9 shows the integrated intensity ratios of the fourth to fifth and fourth to third order A_1_(LO) modes at 160 K and 300 K. With the increase in excitation power, the intensity ratios of the fourth to fifth and fourth to third order A_1_(LO) modes varied with the same tendency at 160 K. Nevertheless, the intensity ratios of the fourth to fifth order A_1_(LO) modes decreased, and the fourth to third order A_1_(LO) modes increased with excitation power at 300 K. The difference between the results at the two temperature points also indicates that thermal activation of another electronic level within the bandgap involved in the electronic transition appeared at 170 K and was responsible for the change in recombination.

**Figure 10:**
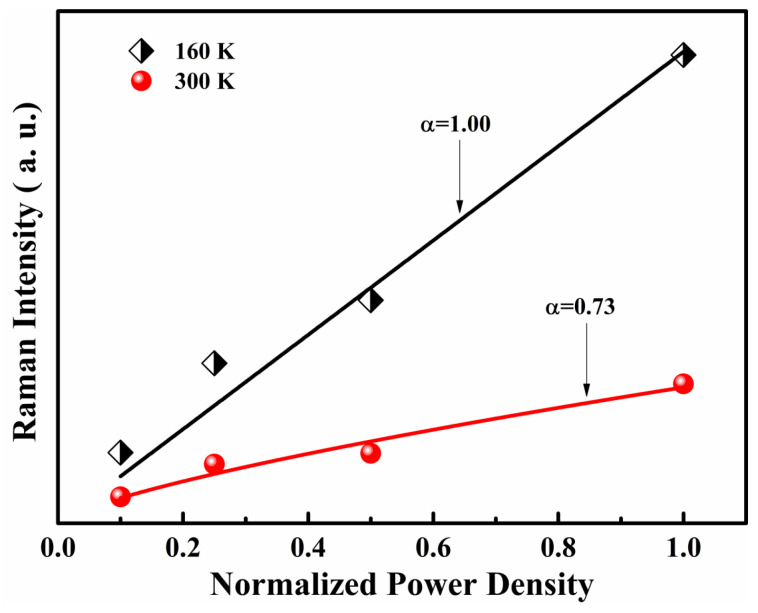
Dependence of the sum of integrated intensities of the first–fifth-order A_1_(LO) modes on excitation power densities at 160 K and 300 K for the rapid thermal annealing treated, B-implanted GaN.

The intensities of the first–fifth A_1_(LO) modes as a function of excitation power at 160 K and 300 K are shown in Figure 10. The equation that describes the relation between the transition intensity (I) and excitation power is as follows:

(5) $$I=CI_{0}^{\alpha }$$

where *α* is the exponent that depends on transition in the sample and *C* is a constant. For an *α* value higher than one, electron-hole recombination occurs from the band edge, i.e., the optical transition is band-edge-related. On the other hand, when α is less than or equal to one, the optical transition is an interband transition [40]. By changing the percentage of excitation powers to 10%, 25%, 50%, and 100%, the α values are estimated to be 1 and 0.73 for 160 K and 300 K, respectively. According to the discussion above, the two values of α derived at two different temperatures indicate that there is at least one donor or acceptor level involved in each of the two transitions, which coincides with the transition energy that we estimated was lower than band-edge transition.

The energy 39–131 meV lower than the band-edge transition indicates that the levels involved in the interband transitions are quite shallow. It indicates that the optical transitions is from one of three origins: band-edge, shallow-donor, or shallow-acceptor transitions. The turning point also suggests the thermal activation of a new electronic level formed within the bandgap.

## 4. Conclusions

In summary, we investigated the evolution of the structural and optical properties of a GaN epilayer grown by MOCVD following B-implantation and RTA processes. The HRXRD revealed that RTA treatment can effectively heal the implantation-induced damage in B-GaN. The optical properties were investigated by RRS measurements based on the positions of the most enhanced modes at different temperatures in the spectra, and we concluded that the dominant transitions changed from band-edge-related to dopant-related. Strong RRS and radiative absorption in GaN resulted in strong PL emission before B-ion implantation. The PL quenched in B-implanted GaN. The inherently weak new transition peaks were identified in the range of 3.357–3.449 eV for B-ion-implanted GaN by combining the experimental data and calculations. Using this approach, two dominant transitions in different temperature ranges were identified that were not band-edge-related transitions. With the assumption that the intensity of the RRS signal detected is linearly proportional to the intensity of the optical emission involved in the RRS process, the activation energies of 66 meV and 116 meV were estimated for two transitions. Power-dependent RRS measurements showed the transition at high temperature has a lower exponential dependence on the excitation intensity compared to the transition at low temperature.

This work demonstrates the strong influence of B-ion implantation on electronic transitions in GaN with very small changes in crystal structure. The work further emphasizes the strength of the RRS technique to explore both the transitional and vibrational properties of a semiconductor. Additionally, an empirical relationship was found that effectively describes the temperature effect in B-GaN.

## Data Availability

The data presented in this study are available on request from the corresponding authors.
